# Cardiac surgical experience in northern Nigeria

**DOI:** 10.5830/CVJA-2012-028

**Published:** 2012-09

**Authors:** J Nwiloh, S Edaigbini, S Danbauchi, M Aminu, A Oyati, I Babaniyi, Y Adamu

**Affiliations:** Section of Cardiothoracic Surgery, St Joseph’s Hospital, Atlanta, Georgia, USA; Ahamadu Bello University Teaching Hospital, Zaria, Nigeria; Ahamadu Bello University Teaching Hospital, Zaria, Nigeria; Ahamadu Bello University Teaching Hospital, Zaria, Nigeria; Ahamadu Bello University Teaching Hospital, Zaria, Nigeria; National Hospital, Abuja, Nigeria; National Hospital, Abuja, Nigeria

**Keywords:** rheumatic, congenital, heart surgery

## Abstract

**Abstract:**

A pilot study was undertaken to determine the feasibility of establishing a heart surgery programme in northern Nigeria. During three medical missions by a visiting US team, in partnership with local physicians, 18 patients with heart diseases underwent surgery at two referral hospitals in the region. Sixteen (88.9%) patients underwent the planned operative procedure with an observed 30-day mortality of 12.5% (2/16) and 0% morbidity. Late complications were anticoagulant related in mechanical heart valve patients and included a first-trimester abortion one year postoperatively, and a death at two years from haemorrhage during pregnancy. This has prompted us to now consider bioprosthetics as the valve of choice in women of childbearing age in this patient population. This preliminary result has further stimulated the interest of all stakeholders on the urgency to establish open-heart surgery as part of the armamentarium to combat the ravages of heart diseases in northern Nigeria.

## Abstract

Northern Nigeria, with over 50% of the nation’s estimated 150 million population, has several tertiary-care hospitals but none has the capacity for open-heart surgery to service the large number of indigent patients affected by the ravages of rheumatic and congenital heart diseases. These patients therefore have a grim prognosis and many face untimely death, with the exception of a minority who have the financial resources or are able to obtain government or private sponsorship to travel abroad for the recommended surgical treatment.

Medical treatment is the only available option and is often palliative, with many patients requiring frequent hospitalisations for congestive heart failure and with a resultant poor quality of life. Because of this dismal outlook, the Global Eagle Foundation, a US-based non-governmental organisation, in partnership with the Nigerian Government, decided to undertake a pilot project on the feasibility of establishing a heart programme to fill this void and bring hope to these patients.

This report summarises our initial experience with the first series of open-heart surgeries ever performed in northern Nigeria.

## Methods

Between October 2006 and April 2008, patients referred with heart diseases to the Cardiology Division of the National Hospital, Abuja and Ahmadu Bello University Teaching Hospital, Zaria, were screened and potential surgical candidates were shortlisted. After further evaluation, patients testing positive for HIV/AIDS and hepatitis B and C were excluded. Due to the limited resources, the more symptomatic patients were selected to undergo surgery.

Diagnosis was established non-invasively through clinical examination and confirmed by transthoracic echocardiogram (TTE). Transoesophageal echocardiogram (TEE) and cardiac catheterisation were not available then at either institution. All the valvular patients met the American College of Cardiology/American Heart Association (ACC/AHA) class I indications for surgery.

During the three missions, each lasting about one week, 18 patients comprising 12 (66.7%) females and six (33.3%) males, with age range five to 42 years (mean 17.6 years), underwent heart surgery. Twelve (66.6%) patients had acquired heart diseases, predominantly rheumatic valvular disease and six (33.3%) had congenital heart disease, of whom 55.5% (10/18) were either in NYHA class 3 or 4 pre-operatively (Table 1).

**Table 1.** Pre-Operative Diagnosis And NYHA Class

*Diagnosis*	*Number of patients*
Acquired heart disease	
Severe mitral regurgitation	7
Severe mitral stenosis	2
Severe aortic regurgitation	2
Penetrating heart wound	1
Congenital heart disease	
Ventricular septal defect	2
Patent ductus arteriosus	2
Atrial septal defect	1
Tetralogy of Fallot	1
NYHA	
Class I	1
Class II	7
Class III	3
Class IV	7

The EURO score was used for risk stratification in the valve patients, with a mean score of 5.51, and range of 3.13–12.04. Invasive monitoring was by arterial and central venous pressure lines, with a swan ganz catheter and cardiac output measurements used sparingly due to limited supply.

Surgical exposure was through a median sternotomy for patients requiring the heart–lung machine. Cardiopulmonary bypass was via ascending aortic and atrial–bicaval cannulations. Moderate systemic hypothermia at 30°C was used in all patients and myocardial protection during aortic cross clamping was by cold-blood cardioplegia administered antegrade, retrograde or both.

Standard blood-conservation techniques used included, whenever possible, retrograde priming of the pump with removal of blood and cell saver. Two patients received low-dose aprotonin. The blood banks did not have the capability to provide component blood therapy and therefore only whole blood was available to transfuse for either anaemia or coagulopathy. Postoperative follow up was by clinic visits or telephone calls and was completed in 87.5% of surviving patients.

## Results

Of the 18 patients undergoing surgery, 16 (88.8%) completed the planned operative procedure. Two patients were deemed inoperable after sternotomy due to supra-systemic pulmonary hypertension, determined by direct-needle transducer measurements. There was no pre-operative swan ganz floated in these two patients as none was available, and the pre-operative TTE had grossly underestimated the pulmonary artery pressures. The operative procedures performed are listed in Table 2.

**Table 2.** Operations Performed

*Operations*	*No of patients*
Acquired heart disease	
Mitral valve replacement	7
Aortic valve replacement	2
Repair right ventricular laceration	1
Congenital heart disease	
Closure ventricular septal defect	2
Ligation patent ductus arteriosus	2
Closure atrial septal defect	1
Modified Blalock-Tausig shunt	1

Time on cardiopulmonary bypass was 20–205 minutes (mean 130) and on aortic cross clamp 89–114 minutes (mean 103). All patients were initially easily weaned off bypass, although two later required intra-aortic balloon pump (IABP) post bypass for haemodynamic instability. Mechanical valves were used for heart valve replacement and comprised four St Judes, three ATS and two On-X valves. Septal defects were closed primarily if less than 1 cm in diameter or otherwise glutaraldehyde-treated autologous pericardium was used. An 8-mm Gore-Tex graft was used for the modified Blalock-Taussig shunt.

Seven patients (43.7%) required blood, with a mean of two units of whole blood transfused. Two patients who received aprotonin were almost bone dry and required no transfusion. All patients were extubated within 12 hours, with the exception of one patient who required ventilation for longer than 24 hours. Anticoagulation with intravenous heparin and Coumadin was started on postoperative day 2 if there was no evidence of significant bleeding. Heparin was discontinued once the INR was within the desired therapeutic range of 2.5 to 3.5.

Thirty-day operative mortality was 12.5% (2/16) and involved the first index cases at both institutions to undergo openheart surgery. Both were females with chronic severe aortic regurgitation from rheumatic heart disease. The first patient had severe pericarditis with dense adhesions, and following uneventful surgery developed sudden pulmonary hypertension and systemic hypotension a few minutes after protamine sulphate administration, which was unresponsive to standard therapeutic measures. Heparin was re-administered and cardiopulmonary bypass quickly reinstituted due to haemodynamic collapse. After a period of rest with an empty, beating heart, the patient was separated from the bypass with inotropes and IABP. She developed coagulopathy and died of haemorrhage, as fresh frozen plasma, platelets and cryoprecipitate were unavailable at the blood bank.

The second patient, also after an uneventful surgery, developed unexplained sudden hypotension while transferring to the intensive care unit and required pressors and IABP for stabilisation. She required prolonged ventilation and died four days later from pneumonia-related sepsis due to unavailability of potent broad-spectrum antibiotics, in addition to a delay in obtaining microbiological laboratory results.

There was no re-operation for bleeding, cardiac tamponade or valvular dysfunction. There was no stroke, renal failure, deep sternal wound infection or any other major morbidity. At follow up there was one anticoagulant-related morbidity one year postoperatively in a valve patient on Coumadin, resulting in a first-trimester abortion, and a late mortality two years postoperatively in the same patient from anticoagulant-related haemorrhage during another pregnancy.

## Discussion

In the 50 years since the introduction of the heart–lung machine to clinical practice by Gibbons in 1953, open-heart surgery has matured as a speciality and become routine in all the developed nations and most of the underdeveloped world. However, sub-Saharan Africa which, according to the World Health Organisation lags behind in most aspects of healthcare, has yet to develop heart surgery programmes to any significant extent. While infectious diseases and malnutrition presently remain their leading public health concerns, cardiovascular diseases are expected to gain more prominence in coming decades.

Although there is a paucity of data on heart diseases in sub-Saharan Africa, the consensus of experts is that rheumatic fever and the sequelae of rheumatic heart disease are the commonest forms of heart disease in Africa, followed closely by dilated cardiomyopathy.^1^-^3^ Both of these diseases affect mainly children and young adults from socio-economically disadvantaged segments of the population living in unsanitary conditions, which predisposes them to infectious diseases such as Group A Streptococcal pharyngitis, compounded by malnutrition as a consequence of poverty.

While the exact incidence of congenital heart disease in the population is unknown, it is estimated to occur in one in 100 live births worldwide. Ischaemic heart disease, prevalent in the industrialised world, is rare in sub-Saharan Africa and seen only in the small segment of the population exposed to Western diet and lifestyles.

Seventy per cent of the estimated 150 million population of Nigeria live below the poverty line, with inadequate housing, sanitation and basic health services; 45.1% of the population is under 15 and 4.8% over 65 years of age, with a life expectancy of 52 years for men and 52.2 years for women.^4^ It is therefore obvious that the number of children and young adults from socio-economically disadvantaged backgrounds exposed to rheumatic fever and the sequelae of rheumatic heart disease is enormous.

Moreover, the incidence is expected to increase in coming decades due to worsening economic conditions in sub-Saharan Africa, combined with the ravages of HIV/AIDS, rendering many children orphans and homeless. Because of lack of heart surgery programmes in most of sub-Saharan Africa, many of these patients with heart diseases and requiring surgery are therefore treated only medically due to the prohibitive cost of travelling abroad for open-heart surgery.

In Nigeria, the first open-heart surgery was performed at the University of Nigeria Teaching Hospital, Enugu (UNTH) in 1974 by a team from the United Kingdom, led by Prof Magdi Yacoub.^5^ The hospital, for years the only cardiac surgery centre in Nigeria, has however remained dormant in recent years.^6^ A second heart programme, also in southern Nigeria, started at the Lagos State University Teaching Hospital, Lagos (LASUTH) in 2004 and is still in its infancy.

In contrast, northern Nigeria with over 50% of the country’s population has no heart surgery programme, despite the large number of patients needing such specialised services. As a result, many of the patients referred to us for surgery had advanced cardiomyopathy with pulmonary hypertension and cardiac cachexia.

One might have expected a higher mortality and morbidity in this pilot study because of the high risk profiles, coupled with the total lack of experience at institutional and personnel levels. However, the results were satisfactory, as the two deaths were potentially avoidable and attributable to lack of needed resources from the blood bank and pharmacy. The observed mortality in this high-risk group was exaggerated by our relatively low numbers. There was of course an obvious learning curve with our index cases, involving all the support services and personnel, which prevented further complications in subsequent patients at both institutions.

Identified deficiencies included lack of blood bank capability to provide component blood therapy, which contributed to the death of our first patient from severe coagulopathy, and the unavailability of potent broad-spectrum antibiotics from the pharmacy, contributing to the second mortality from nosocomial infection. Furthermore upon realising the total absence of respiratory therapist support and the less-than-ideal sterility of the ventilatory tubing which was reused for multiple patients, all efforts were made for early extubation within a few hours of surgery to reduce the risk of cross contamination of the respiratory tract.

This strategy required coordination by the anesthesiologists and perfusionist, with the use of easily reversible anaesthetic agents and keeping patients dry with ultrafiltration on bypass to reduce lung water which might affect lung compliance postoperatively. We believe that this strategy of early extubation and mobilisation, along with the relatively young age of the patients may have contributed to the absence of any major morbidity in the surviving patients.

The late morbidity and mortality at one and two years, respectively, were both anticoagulant-related haemorrhage, in a pregnant woman with a mechanical valve, and poor compliance with Coumadin monitoring. Because of the higher risk of valve calcification in the young and the cost of a possible future re-operation for structural valve deterioration, all the patients had opted for a mechanical valve, although some were already on anticoagulation for chronic atrial fibrillation.

The risk of thromboembolism and haemorrhage is estimated at about 2% per year, even in the best setting, and this figure is likely to be even higher in our impoverished population with inadequate anticoagulation monitoring. The cumulative risk for thromboembolic and haemorrhagic complications in these patients over a lifetime is therefore enormous and has prompted us to reconsider the use of mechanical valve replacement in this largely poor and uneducated population, most especially in females of childbearing age. This is particularly important as rheumatic heart disease in northern Nigeria appears more common in females, as reported by Danbauchi *et al*.^3^ We therefore now recommend bioprosthetic valves to females of childbearing age in this patient population.

Because of poverty and lack of education, monitoring of adequate levels of anticoagulation can be challenging, if not impossible, especially for those living in remote villages that are unable to follow up regularly at the clinics. Due to lack of standardisation and quality control, the results, even in those undergoing regular monitoring, are sometimes unreliable and inconsistent, perhaps due to the use of expired reagents in some of the laboratories.

## Conclusion

Despite the fact that most of the patients had advanced cardiomyopathy and were often malnourished, the overall outcome was excellent considering that this was the first series of heart surgeries in this region, performed in less-than-ideal operating conditions, including lack of equipment and ancillary support services. The two operative mortalities were potentially avoidable had adequate support structures been in place, and also represented a learning curve for this type of delicate surgery at both institutions. These initial results are however encouraging and show that with adequate government financial support for equipment acquisition and human capacity building, northern Nigeria should be able to support two heart surgery programmes to service this large patient population.
